# The role of NLRP3 inflammasome in hepatocellular carcinoma

**DOI:** 10.3389/fphar.2023.1150325

**Published:** 2023-04-20

**Authors:** Huijie Zhao, Yiming Zhang, Yanting Zhang, Chaoran Chen, Huiyang Liu, Yihan Yang, Honggang Wang

**Affiliations:** ^1^ Institute of Chronic Disease Risks Assessment, Henan University, Kaifeng, China; ^2^ Institute of Nursing and Health, School of Nursing and Health, Henan University, Kaifeng, Henan, China; ^3^ School of Clinical Medicine, Henan University, Kaifeng, Henan, China; ^4^ School of Basic Medical Sciences, Henan University, Kaifeng, Henan, China

**Keywords:** hepatocellular carcinoma, NLRP3 inflammasome, pyroptosis, apoptosis, reactive oxygen species

## Abstract

Inflammasomes play an important role in innate immunity. As a signal platform, they deal with the excessive pathogenic products and cellular products related to stress and injury. So far, the best studied and most characteristic inflammasome is the NLR-family pyrin domain-containing protein 3(NLRP3) inflammasome, which is composed of NLRP3, apoptosis associated speck like protein (ASC) and pro-caspase-1. The formation of NLRP3 inflammasome complexes results in the activation of caspase-1, the maturation of interleukin (IL)-1β and IL-18, and pyroptosis. Many studies have demonstrated that NLRP3 inflammasome not only participates in tumorigenesis, but also plays a protective role in some cancers. Hepatocellular carcinoma (HCC) is a major cause of cancer-related mortality. Currently, due to the lack of effective treatment methods for HCC, the therapeutic effect of HCC has not been ideal. Therefore, it is particularly urgent to explore the pathogenesis of HCC and find its effective treatment methods. The increasing evidences indicate that NLRP3 inflammasome plays a vital role in HCC, however, the related mechanisms are not fully understood. Hence, we focused on the recent progress about the role of NLRP3 inflammasome in HCC, and analyzed the relevant mechanisms in detail to provide reference for the future in-depth researches.

## 1 Introduction

**FIGURE 1:**
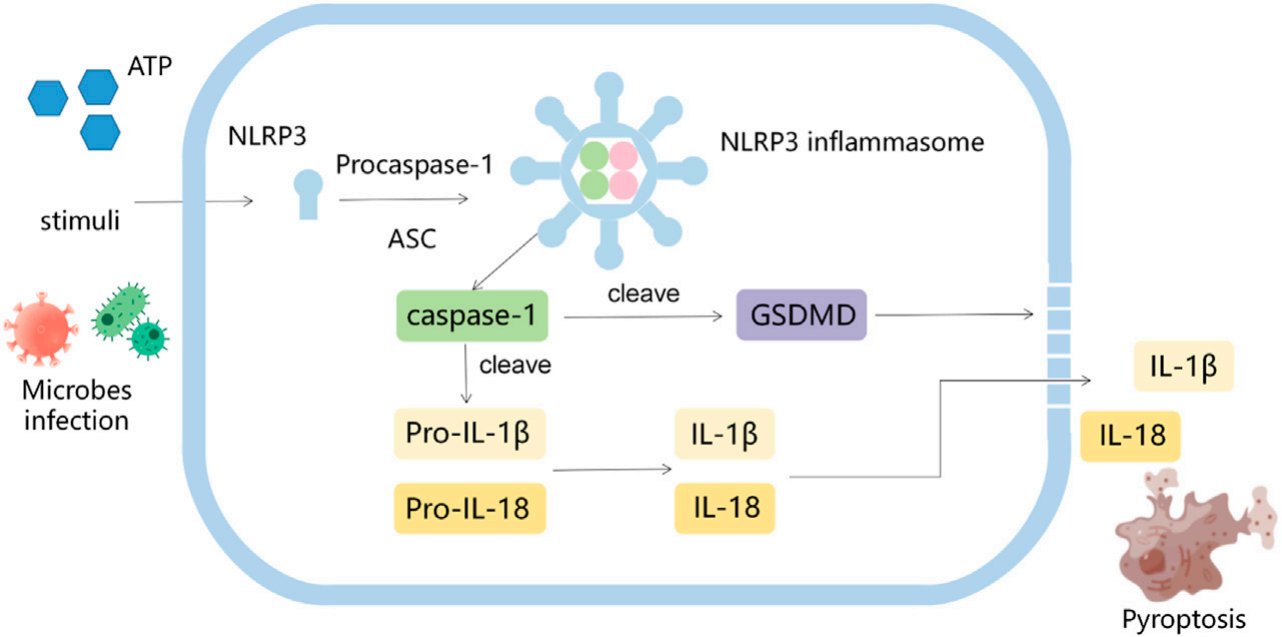
The diagram of activation process of NLRP3.

Inflammasomes, firstly proposed by Tschopp et al., in 2002, are multi-protein complexes that can activate procaspase-1 in response to “damaged-self” signals or infection (Hoffman et al., 2001; Martinon et al., 2002; Martinon et al., 2002; Broz and Dixit, 2016; Collison, 2019). The activated caspase-1 in turn transforms pro-IL-1β and pro-IL-18 into their mature forms to induce inflammation (Broz and Dixit, 2016). A variety of inflammasomes have been found: NLR-family pyrin domain-containing protein 1(NLRP1), NLRP2, NLRP3, NLRP6, NLRP7, NLRP12, NLRC4, IPAF and AIM2. Among them, NLRP3 inflammasome is the most thoroughly studied one, consisting of NLRP3, apoptosis associated speck like protein (ASC) and pro-caspase-1 (Volt et al., 2016; Sharif et al., 2019). Pathogen-associated molecular patterns (PAMPs) (including microorganisms, misfolded proteins, crystals and nanoparticles) and damage-associated molecular patterns (DAMPs) (such as extracellular ATP), induce the formation of NLRP3 oligomers, and the recruitment of ASC and pro-caspase-1, resulting in the activation of caspase-1 and the cleavage of gasdermin D (GSDMD). The activated caspase-1 cleaved pro-IL-1β and pro-IL-18 into their active forms. The N-terminal domain of cleaved GSDMD drills pores in the cell membrane and promotes the release of IL-1β and IL-18, thus inducing pyroptosis (Figure 1) (Ding et al., 2021; Coll et al., 2022). The evidences indicate that NLRP3 inflammasome is closely related to the tumorigenesis, including proliferation, invasion, angiogenesis and metastasis (Moossavi et al., 2018; Ershaid et al., 2019; Sharma and Kanneganti, 2021), however, the relevant mechanisms are not completely clear.

Liver cancer is the leading cause of cancer-related deaths and a major health problem in the world. There are more than 850,000 cases of liver cancer every year (Forner et al., 2018; Sim and Knox, 2018). Hepatocellular carcinoma (HCC) is the most common tumor and accounts for the vast majority of primary liver cancers (Ganne-Carrié and Nahon, 2019; Lurje et al., 2019). The incidence rate of HCC related to non-alcoholic fatty liver disease (NAFLD) and non-alcoholic steatohepatitis (NASH) is increasing. It is estimated that by 2030, the global mortality rate of HCC will reach 1 million people per year (Fitzmaurice et al., 2017; Anstee et al., 2019). The occurrence of HCC is related to many factors, including alcoholism, cirrhosis (chronic liver damage caused by fibrosis), hepatitis B virus (HBV) infection, hepatitis C virus (HCV) infection and metabolic syndrome (Piñero et al., 2020). The pathogenesis of HCC is complex, including cell cycle disorder, immune regulation disorder, microRNA (miRNA) disorder, DNA methylation change, chromosome instability, epithelial-to-mesenchymal cell transformation (EMT) and the increase of HCC stem cells (Chidambaranathan-Reghupaty et al., 2021). HCC stem cells have many characteristics similar to those of normal liver stem cells. In addition to self-renewal and tumorigenesis, it is also associated with patient resistance to treatment and relapse (Ogunwobi et al., 2019). At present, the treatment schemes for HCC include surgical resection, liver transplantation and minimally invasive local treatment including percutaneous ablation, transcatheter arterial chemoembolization (TACE) and transcatheter arterial radiation embolism (TARE) (Tellapuri et al., 2018). Patients diagnosed in the late stage of the disease are not eligible for therapeutic surgery, and treatment options for patients with advanced HCC are limited in terms of availability and effectiveness. In order to improve the status of early diagnosis and treatment, the better understanding of molecular biology of HCC is very needed (Kim and Viatour, 2020). More and more evidence indicates that NLRP3 inflammasome plays an important role in HCC, however, the relevant mechanisms are needed to be further studied. In this review, we summarized the literatures on the role of NLRP3 in HCC in recent years, and deeply analyzed the relevant mechanisms, hoping to reveal the role of NLRP3 inflammasome in HCC and provide theoretical reference for further researches in the future.

## 2 NLRP3 inflammasome-mediated pyroptosis plays a protective role in HCC

It has been reported that 17β-estradiol (E2) is involved in HCC via promoting the NLRP3 inflammasome activation (Wei et al., 2015), however, the related mechanisms are not fully understood. Qing Wei and others found that E2 promoted NLRP3 inflammasome activation through increasing the expression levels of caspase-1 and IL-1β in HCC cells. Meanwhile, E2 also decreased the viability of HCC cells and increased HCC cells mortality rate. The caspase-1-specific inhibitor YVAD-cmk significantly abolished the E2-induced inhibition of growth of HCC cells effect, suggesting that E2 induced the death of HCC cells by promoting NLRP3 inflammasome-caspase-1 dependent pyroptosis. The in-depth experiments revealed that E2 significantly inhibited autophagy in HCC cells, which was abolished by YVAD-cmk, indicating that NLRP3 inflammasome mediated E2-induced inhibition of autophagy. E2 inhibited AMPK/mTOR pathway by downregulating AMPK phosphorylation level and upregulating mTOR phosphorylation level, and AMPK overexpression and rapamycin (an mTOR inhibitor) reversed E2-induced autophagy inhibition, suggesting that E2 suppressed autophagy by inhibiting AMPK/mTOR pathway. In addition, 3-MA, an autophagy inhibitor significantly upregulated E2-induced pyroptosis, which was reversed by YVAD-cmk, suggesting that autophagy negatively regulated inflammasome-caspase-1 dependent pyroptosis induced by E2. Collectively, E2 induced NLRP3 inflammasome-caspase-1 dependent pyroptosis by suppressing autophagy through inhibiting AMPK/mTOR pathway in HCC cells. E2-induced NLRP3 inflammasome promoted HCC via upregulating caspase-1-dependent pyroptosis of HCC cells (Wei et al., 2019). The interaction between NLRP3 inflammasome and autophagy participates in many pathological processes (Tao et al., 2020; Ding et al., 2021; Lv et al., 2021). In the above study, autophagy inhibition promotes E2-induced NLRP3 inflammasome of HCC cells, thus providing a new idea for HCC treatment by regulating autophagy. Conversely, E2-induced autophagy can also be negatively regulated by NLRP3 inflammasome in HCC cells. Therefore, the relationship between NLRP3 inflammasome and autophagy in HCC deserves further study in the future, which will help to explore the pathogenesis of HCC.

Another study has confirmed the above conclusion that promoting NLRP3 inflammasome-mediated pyropsis significantly inhibits HCC. Alpinumisoflavone (AIF) is a dimethyl pyran derivative that is isoprenoized on the ring A of genistein. It is the main component of Derris eriocarpa F.C. (Ateba et al., 2019). AIF has been reported to have a variety of pharmacological activities, including estrogenic (Mvondo et al., 2012), atherosclerosis protection (Ateba et al., 2019), estrogen and antibacterial (Chukwujekwu et al., 2011). In recent years, AIF has been found to have potential anti-tumor effects (Zhang et al., 2021). However, the relevant mechanisms are not yet fully understood. Yan Zhang and others studied the role of AIF and pyroptosis in HCC, and the results showed that AIF could inhibit the growth and clonogenic capacity of HCC cells, but not normal hepatocytes. In HCC cells treated with AIF, the number of HCC cells with invasion and migration ability was significantly reduced, the protein expression levels of matrix metalloproteinase-2 (MMP-2) and matrix metalloproteinase-9 (MMP-9) were significantly reduced, while the expression level of metalloproteinases-3 (TIMP3) was significantly increased, indicating that AIF inhibited the migration and invasion of HCC cells. The in-depth research showed that AIF increased the release of lactate dehydrogenase (LDH), and upregulated the expression levels of NLRP3, caspase- 1, IL-18 and IL-1β, suggesting that AIF induced the pyroptosis of HCC cells through activating NLRP3-caspase-1-IL-1β pathways. The treatment of MCC950 (NLRP3 inflammasome inhibitor) abolished the effect of AIF on pyroptosis-related genes. Moreover, NLRP3 knockdown inhibited AIF-induced increase of cleaved caspase-1, mature IL-1β, IL-18, and GSDMD-N, and weakened the inhibitory effects of AIF on the viability and motility of HCC cells. These results indicated that AIF-induced pyroptosis of HCC cell was dependent on the activation of NLRP3, and AIF inhibition of the viability and motility of HCC cells was mediated by pyroptosis. Furthermore, AIF treatment enhanced autophagy by upregulating the expression levels of the autophagy-related proteins including LCII, and beclin 1 and downregulating p62 expression in HCC cells. Further, CQ (autophagy inhibitor) or *Atg* siRNA promoted AIF-induced NLRP3 inflammasome-mediated pyroptosis of HCC cells, which seemed to contradict with the above that AIF promoted autophagy. Does AIF-upregulated autophagy inhibit AIF-induced NLRP3 inflammasome? The reason needed to be clarified. Collectively, AIF inhibited the growth and metastasis of HCC cells by inducing NLRP3 inflammasome-mediated pyroptosis, (Zhang et al., 2020). In the above study, the relationship between autophagy and NLRP3 inflammasome in HCC cells deserves further study in the future. In addition, it has been reported that AIF can promote the apoptosis of cancer cells (Han et al., 2016; Hong et al., 2022). Therefore, whether AIF can induce apoptosis of HCC cells through NLRP3 inflammasome remains to be clarified.

Estrogen mediates the various systemic effects in women and men, and regulates the physiological and pathological processes of reproductive, skeletal, nervous, cardiovascular, endocrine and immune systems (Tang et al., 2019). The effect of estrogen is mainly mediated by estrogen receptor (ER)ɑ and ERβ (Song et al., 2019). It has been reported that 17β-estradiol (E2, a form of estrogen) or ERβ is invovled in HCC (Teng et al., 2014; Ren et al., 2016; Xu et al., 2018). However, the role and mechanism of E2 and ERβ in HCC have not been clarified. Qing Wei and others found that compared with normal liver tissue, the ERβ expression in HCC tissue was notably decreased. Furthermore, the protein level of ERβ was negatively correlated with the pathological grades and clinical stages of the HCC patients, and positively correlated with the levels of NLRP3 inflammasome components. In addition, E2 significantly increased the expression of NLRP3 inflammasome, and activated MAPK/ERK pathway in HCC cells. The ERβ-specific inhibitor or the inhibitor of MAPK pathway could abolish E2-induced increase of the expression of NLRP3 inflammasome, indicating that E2 upregulated the expression of NLRP3 inflammasome through activating ERβ/MAPK/ERK pathway. The experiments using HCC cells transfected with the plasmids encoding NLRP3 inflammasome demonstrated that the levels of NLRP3 inflammasome, caspase-1 and IL-1β were significantly increased, the proliferation of these HCC cells was inhibited, and the LDH release was upregulated, indicating that overexpression of NLRP3 suppressed the growth of HCC cells and promoted the death of HCC cells. Moreover, the inhibition of ERβ/MAPK/ERK pathway abolished E2 inhibition of the cell viability, colony formation capability and migration of HCC cells, suggesting that E2 suppressed HCC by activating NLRP3 inflammasome via ERβ/MAPK/ERK pathway (Wei et al., 2015). The above conclusions well explain why the incidence of HCC has gender differences. The promotion of pyroptosis and apoptosis has been reported to inhibit HCC (Wan et al., 2018; Shen et al., 2021; Yan et al., 2022). Therefore, from the above, it can be deduced that E2 suppressed HCC through promoting NLRP3 inflammasome-mediated pyroptosis (Wei et al., 2015).

## 3 NLRP3 inflammasome-mediated apoptosis plays a protective role in HCC

MicroRNAs (miRNAs) are a group of highly conserved non-protein-coding small RNAs, which are post-transcriptional regulators of gene expression. Many studies have shown that miRNA can regulate many biological processes, including cancer, inflammation and metabolism (Wurdinger and Costa, 2007; Quintanilha et al., 2017). MiR-223-3p has been reported to interact with the 3′-untranslated region fragment of NLRP3 and suppress its expression (Deng et al., 2020; Wang et al., 2021). In addition, many evidences indicate that miR-223-3p is involved in HCC (Pratedrat et al., 2020; Si et al., 2021). However, the role and mechanism of miR-223-3p in regulating NLRP3 in HCC have not been understood. To clarify this, LINGFENG WAN and others committed a series of experiments and MTT assay showed that the proliferation of HCC cells transfected with miR-223-3p decreased significantly. At the same time, after transfection with miR-223-3p, the apoptosis rate increased significantly. These results indicated that miR-223-3p had antitumor effect on HCC cells. The dual-luciferase reporter assay showed that the co-transfection of miR-223-3p decreased luciferase activity of the plasmid containing the fragment of NLRP3 3′-UTR. However, the luciferase activity of the plasmid containing the mutant fragment of NLRP3 3′-UTR was not influenced by co-transfection with the miR-223 analog, indicating that miR-223-3p directly interacted with 3′- UTR of NLRP3 mRNA.Further experiments showed that after transfection with miR-223-3p, the expression of NLRP3, caspase-1, IL-1β and IL-18 in HCC cells was inhibited, indicating that miR-223-3p suppressed NLRP3 inflammasome. Moreover, the inhibition of NLRP3 inflammasome, IL-1β and IL-18 promoted the apoptosis of HCC cells. Collectively, miR-223-3p inhibited NLRP3 inflammasome to inducing apoptosis, thus inhibiting the proliferation of HCC cells (Wan et al., 2018). At present, it has been reported that NLRP3 inflammasome positively regulates apoptosis of cancer cells (Jabir et al., 2021), which is inconsistent with the conclusion that inhibition of NLRP3 inflammasome induces apoptosis of HCC cells in above studies. Therefore, the relationship between NLRP3 inflammasome and apoptosis, especially in cancer, needs further study.

Anisodamine (ANI) is a belladonna alkaloid. Like other drugs in the family, it is not only a nicotine choline receptor antagonist, but also a non-subtype‐selective muscarine (Zhao et al., 1993). It has been reported that ANI also has a significant anti-inflammatory effect, which can reduce the damage of kidney and heart by inhibiting the activation of inflammasomes and decreasing the expression levels of inflammatory factors. However, the role of ANI in cancers has not yet been clarified (Yuan et al., 2017; Yao et al., 2018). ANI can alleviate the tissue damage in diseases by regulating NLRP3 inflammasome (Yuan et al., 2017; Li et al., 2020a). However, the role of ANI in regulating NLRP3 in HCC is unclear. Ping Li et al. studied the molecular mechanism of the anti-cancer role of ANI in HCC through inhibiting NLRP3 inflammasome by constructing a xenotransplantation mouse model of HCC. The results showed that ANI could inhibit the formation of tumor, increase the survival rate of HCC xenograft mice, and reduce the levels of TG, TC, HDL and LDL in serum. In addition, The results of HE staining showed that ANI treatment improved the histopathological damage of HCC xenotransplantation mice in a dose-dependent manner. These findings indicated that ANI could improve HCC. Moreover, NLRP3 inflammasome was inhibited by ANI evidenced by the decreased levels of ASC, caspase-1, IL-18 and IL-1β. Further analysis showed that NLRP3 overexpression resulted in the increase of tumor volume and decreased survival rate of HCC xenograft mice, indicating that NLRP3 overexpression counteracted the therapeutic effects of ANI on HCC. In-depth mechanism studies showed that ANI reduced the expressions of Ki67 (the proliferating cell related antigen) and vascular endothelial-derived growth factor (VEGF), and induced HCC cells apoptosis, which were reversed by NLRP3 overexpression, indicating that ANI inhibited the growth and motility of HCC cells and induced apoptosis by inhibiting NLRP3 inflammasome. In addition, ANI increased the levels of INF-γ and IL-27, and reduced the levels of TNF-α and IL-4, while overexpression of NLRP3 counteracted this effect. Furthermore, overexpression of NLRP3 reversed ANI-induced reduction in the number of Th1 and Th2 cells. These results indicated that ANI could achieve anti-inflammatory effect to play anti-cancer roles by inhibiting the expression of NLRP3 inflammasome. Furthermore, the knocking down NLRP3 with shRNA enhanced the ANI inhibition of the development of HCC in xenotransplantation mice. Summarily, ANI promoted apoptosis of HCC cells through inhibiting NLRP3 inflammasome (Li et al., 2020b). At present, the relationship between Th1/Th2 balance and cancer has been studied extensively (Shahid and Bharadwaj, 2019; Hetland et al., 2020). In the above study, ANI reduced the number of Th1 and Th2 cells by inhibiting NLRP3 inflammasome (Li et al., 2020b). Therefore, whether ANI can regulate the balance of Th1/Th2 via NLRP3 inflammasome in HCC needs to be further explored. Besides, that ANI inhibits the metastasis of HCC through reducing VEGF expression by inhibiting NLRP3 inflammasome needs further validation.

## 4 NLRP3 inflammasome inhibition enhanced HCC sensitivity to the cytotoxicity of natural killer cells

Natural killer (NK) cells are important natural immune cells which can kill infected viruses and cancer cells (Lim et al., 2015; Fang et al., 2018; Sung and Jang, 2018), and are involved in the first immune defense against viral infection and cancer (Liu et al., 2018). NK cells play an important role in HCC (Guillerey et al., 2016). Although there have been many studies on the role of NK cells in HCC, the role and mechanism of NK cells and NLRP3 inflammasome in HCC are still unclear. Hwan Hee Lee et al. deleted NLRP3 gene in HCC using lentivirus CRISPR-cas9 system, then studied how NLRP3 KO influenced the cytotoxicity of NK cells in HCC. The results showed that NK cytotoxicity to HCC cells lacking NLRP3 was significantly increased. NK cytotoxicity is affected by its NK-activating receptors (NKG2D). The expression of NKG2D on NK cells was notably upregulated in a co-culture of NK cells and NLRP3 KO HCC cells (Lee et al., 2021). It has been reported that NK-activating receptors interact with various ligands on the surface of cancer cells, thereby inducing the toxicity of NK cells to cancer cells (Thompson et al., 2017). MICA/B is a ligand expressed in many cancer cells, which can bind to the NKG2D receptor on NK cells to increase cytotoxicity. Its expression was found to be upregulated on the surface of NLRP3 KO HCC cells through the downregulation of the expression of matrix metalloproteinase in liver tissues of mice implanted with NLRP3 KO HCC cells. Moreover, in a xenograft mice model, NLRP3 KO HCC cells inhibited HCC development and metastasis, and increased HCC sensitivity to the cytotoxicity of NK cells. Collectively, NLRP3 deficiency in HCC increased the cytotoxicity of NK cells to HCC via the interaction of NKG2D-MICA, thus enhancing the immunosurveillance of NK cells (Lee et al., 2021). The immunosurveillance of NK cells is important for HCC aggressiveness (Wang et al., 2016; Cadoux et al., 2021). In the above study, the deletion of NLRP3 inflammasome can enhance the immunosurveillance of NK cells on HCC, which clarifies a new mechanism for the role of NLRP3 inflammasome in HCC. However, the further research is needed on this issue. For example, how NLRP3 inflammasome deficiency upregulates the NK-activating receptors on NK cells and corresponding ligands on HCC cells.

## 5 The role of reactive oxygen species (ROS)/NLRP3 inflammasome in HCC

Luteoloside (luteolin-7-O-glucoside; cymaroside), an active ingredient isolated from Reseda odorata L., has the effects of anti-tumor, anti-inflammatory, antibacterial and free radical scavenging (Baskar et al., 2011; Sun et al., 2011; Xiong et al., 2013). Although the role of luteolide in cancer has been widely studied (hao et al., 2018; Zhou et al., 2017), the mechanism of action is still unclear, especially in HCC. The results of Shao hua Fan and others showed that luteoloside inhibited the proliferation, migration and invasion of HCC cells *in vitro*. Luteoloside has no significant effect on the expressions of caspase-3, IL-CII and Beclin 1 in HCC cells, indicating that luteoloside has no effect on inducing apoptosis and autophagy of HCC cells. Further mechanism studies showed that luteoloside could reduce the accumulation of intracellular ROS, enhance the inhibition of ROS induced by NAC (a specific ROS inhibitor), and reverse the upregulation of ROS induced by H_2_O_2_ (a ROS inducer). In addition, luteoloside downregulated the expressions of NLRP3 inflammasome and IL-1β in HCC cells. *In vivo*, experiments also verified that luteoloside inhibited the proliferation and metastasis of HCC (Fan et al., 2014). Previous studies have revealed that NLRP3 inflammasome can positively regulate the proliferation and metastasis of tumor cells (Ershaid et al., 2019; Hofbauer et al., 2021), and the activation of NLRP3 inflammasome depends on the production of ROS (Li et al., 2021). Therefore, it could be deduced that luteoloside inhibits the proliferation and metastasis of HCC cells by inhibiting NLRP3 inflammasome through reducing intracellular ROS (Fan et al., 2014). In the above study, luteoloside has no signifcant effect on apoptosis and autophagy of HCC cells, indicating that aopoptosis and autophagy-related cell death are not involved in luteoloside inhibition of HCC. Therefore, the mechanism of NLRP3 inflammasome inhibiting the proliferation and metastasis of HCC cells needs to be clarified. The evidences indicate that autophagy, NLRP3 inflammasome and apoptosis are interrelated and play an important role in cancer (Jabir et al., 2021). While in the above study, luteoloside only inhibits NLRP3 inflammasome, but has no effect on apoptosis and autophagy of HCC cells. This indicates that NLRP3 inflammasome appears to be unrelated to apoptosis and autophagy in HCC cells, which contradicts the previous research conclusions. The reason may be related to different types of tumor cells and needs to be clarified.

Shuanghua decoction (SHD) consists of Oldenlandia diffusa Willd., Prunella vulgaris L., Panax ginseng C. A., Meyer, and *Lonicera japonica* Thunb., and is a traditional folk prescription in China. It has been reported that SHD plays an anti-tumor role through suppressing the growth of tumor cells and promoting the apoptosis of tumor cells, and strengthens the immunity (Zhu et al., 2018; Wang et al., 2019a; Ge et al., 2019; Luo et al., 2020; Zhao et al., 2020). The anticancer mechanism of SHD is far from clear. Bingling Dai and others studied the role and mechanism of SHD and NLRP3 inflammasome in HCC, and the results showed that two ingredients of SHD, Oldenlandia and OP (Oldenlandia 15 g: Prunella spike 1.5 g = 10:1), significantly inhibited the growth of HCC cells and xenograft tumors evidenced by the decreased size and weight of tumour, and downregulated Ki67. Oldenlandia could inhibit the colony formation of HCC cells, make more cells stop at S phase of the cell cycle, and increase the release of LDH. Furthermore, Oldenlandia significantly induced apoptosis of HCC cells by increasing the levels of Bax and cleaved PARP, and decreasing Bcl-2 level. In addition, Oldenlandia and OP significantly inhibited the migration of HCC cells. Further studies showed that Oldenlandia upregulated the expressions of NLRP3 inflammasome, pro-IL-1β and cleaved-caspase-1 in a concentration-dependent manner. Oldenlandia also significantly enhanced the activation of NLRP3 inflammasome induced by LPS + ATP. Mechanism studies revealed that Oldenlandia increased ROS release in a concentration and time-dependent manner, while NAC (a ROS scavenger) reduced Oldenlandia-induced ROS release, and weakened the activation of Oldenlandia-induced NLRP3 inflammasome activation, indicating that Oldenlandia activated NLRP3 inflammasome in HCC cells by promoting ROS release. In addition, Oldenlandia reduced IKKβ expression and inhibited LPS (NF-κB activator)-induced phosphorylation of IkBα and NF-κB. PDTC and MG132 (two NF-κB inhibitors) reduced NF-κB phosphorylation, while the combination of Oldenlandia, PDTC and MG132 enhanced the inhibition of NF-κB phosphorylation. These indicated that Oldenlandia inhibited NF-κB pathway. OP could obtain the similar results as Oldenlandia, and also inhibit the expressions of N-cadherin, snail, MMP9, MMP2 and vimentin. Summarily, it can be deduced that SHD activates NLRP3 inflammasome through promting ROS release and inhibiting NF-κB signal pathway to induce apoptosis and cell cycle arrest of HCC cells *in vitro* and *in vivo*, which need to be further confirmed by using the inhibitor of NF-κB signal pathway (Dai et al., 2022). NLRP3 inflammasome-mediated pyroptosis has been reported to play an important role in HCC (Zhang et al., 2020). Whether NLRP3 inflammasome-mediated pyroptosis is involved in SHD inhibition of HCC needs to be studied in the future. ROS/NLRP3 inflammasome will provide an important target for the treatment of HCC.

## 6 Conclusion

In recent years, the increasing evidences show that NLRP3 inflammasome plays an important role in HCC. In this review, we summarize the role of NLRP3 inflammasome in HCC as follows: 1)E2 promotes NLRP3 inflammasome-caspase-1 dependent pyroptosis through inhibiting autophagy by suppressing AMPK/mTOR pathway in HCC cells; 2)AIF inhibits the growth and metastasis of HCC cells by inducing NLRP3 inflammasome-mediated pyroptosis via the inhibition of autophgy; 3)E2 inhibits HCC through promoting NLRP3 inflammasome via activating ERβ/MAPK/ERK pathway; 4)miR-223-3p suppresses NLRP3 inflammasome to induce apoptosis, thus inhibiting the proliferation of HCC cells; 5)ANI promotes apoptosis of HCC cells by suppressing NLRP3 inflammasome; 6)*NLRP3* deficiency in HCC enhances the cytotoxicity of NK cells to HCC via the interaction of NKG2D-MICA, thus promoting the immunosurveillance of NK cells; 7) Luteoloside suppresses the proliferation and metastasis of HCC cells by inhibiting NLRP3 inflammasome via decreasing the intracellular ROS; 8)SHD activates NLRP3 inflammasome through promoting ROS release and suppressing NF-κB pathway, thus inducing apoptosis and cell cycle arrest of HCC cells (Table 1). Our previous research team showed that exogenous H_2_S can regulate NLRP3 inflammasome through AMPK/mTOR pathway (Wang et al., 2019b). In this review, in addition to AMPK/mTOR pathway, ERβ/MAPK/ERK pathway and NF-κB pathway are also involved in the role of NLRP3 inflammasome in HCC. Whether there are other pathways involved in the role of NLRP3 inflammasome in HCC remains to be clarified. The purinergic ligand gated ion channel 7 receptor (P2X7 receptor) is an adenosine triphosphate (ATP) gated ion channel that is widely distributed in various tissues and cells, including HCC cells (Li et al., 2023). The activation of P2X7 receptor can promote the assembly of NLRP3 inflammasome, thereby activating NLRP3 inflammasome (Di Virgilio et al., 2017). The evidence indicates that melatonin inhibits the activation of NLRP3 inflammasome in a mouse model of non-alcoholic steatohepatitis induced by high fat diet by inhibiting P2X7R receptors (Saha et al., 2022). Hence, whether the inhibition of NLRP3 inflammasome via P2X7R receptors can improve HCC is worth studying in the future.

**TABLE 1:** The role of NLRP3 inflamasome in hepatocellular carcinoma.

The role of autophagy and pyroptosis	Experimental model	Reference
E2 promotes NLRP3 inflammasome-caspase-1 dependent pyroptosis through inhibiting autophagy by suppressing AMPK/mTOR pathway in HCC cells	HepG2 hepatoma cell line	Wei et al. (2019)
AIF inhibits the growth and metastasis of HCC cells by inducing NLRP3 inflammasome-mediated pyroptosisvia the inhibition of autophgy	Hepatocellular carcinoma cell lines (humans):HepG2, SMMC 7721, Huh7, Bel7402	Zhang et al. (2020)
E2 inhibits HCC through promoting NLRP3 inflammasome via activating ERβ/MAPK/ERK pathway	human primary HCC samples and human HCC cell lines, including BEL7402, SMMC7721, and HepG2 cells	Wei et al. (2015)
miR-223-3p suppresses NLRP3 inflammasome to induce apoptosis, thsu inhibiting the proliferation of HCC cells	Hep 3B2.1-7 cell line	Wan et al. (2018)
ANI promotes apoptosis, by suppressing NLRP3 inflammasome	HepG2 hepatoma cell line and HCC model of mice	Li et al. (2020b)
NLRP3 deficiency in HCC enhances the cytotoxicity of NK cells to HCC via the interaction of NKG2D-MICA, thus promoting the immunosurveillance of NK cells	NK cell line NK-92, human HCC cells and HCC model of mice	Lee et al. (2021)
luteoloside suppresses the proliferation and metastasis of HCC cells by inhibiting NLRP3 inflammasome via decreasing the intracellular ROS	human HCC cell lines (Hep3B and SNU-449) and HCC model of mic	Fan et al. (2014)
SHD activates NLRP3 inflammasome through promoting ROS release and suppressing NF-κB pathway, thus inhibit the proliferation and migration of HCC cells *in vitro* and *in vivo*	HCC cell lines (SMMC-7721, Bel-7402, Bel-7404, Hep3B, HepG2, Huh7, SK-Hep-1, MHCC-97H, and MHCC-97) and HCC model of mice	Dai et al. (2022)

**FIGURE 2:**
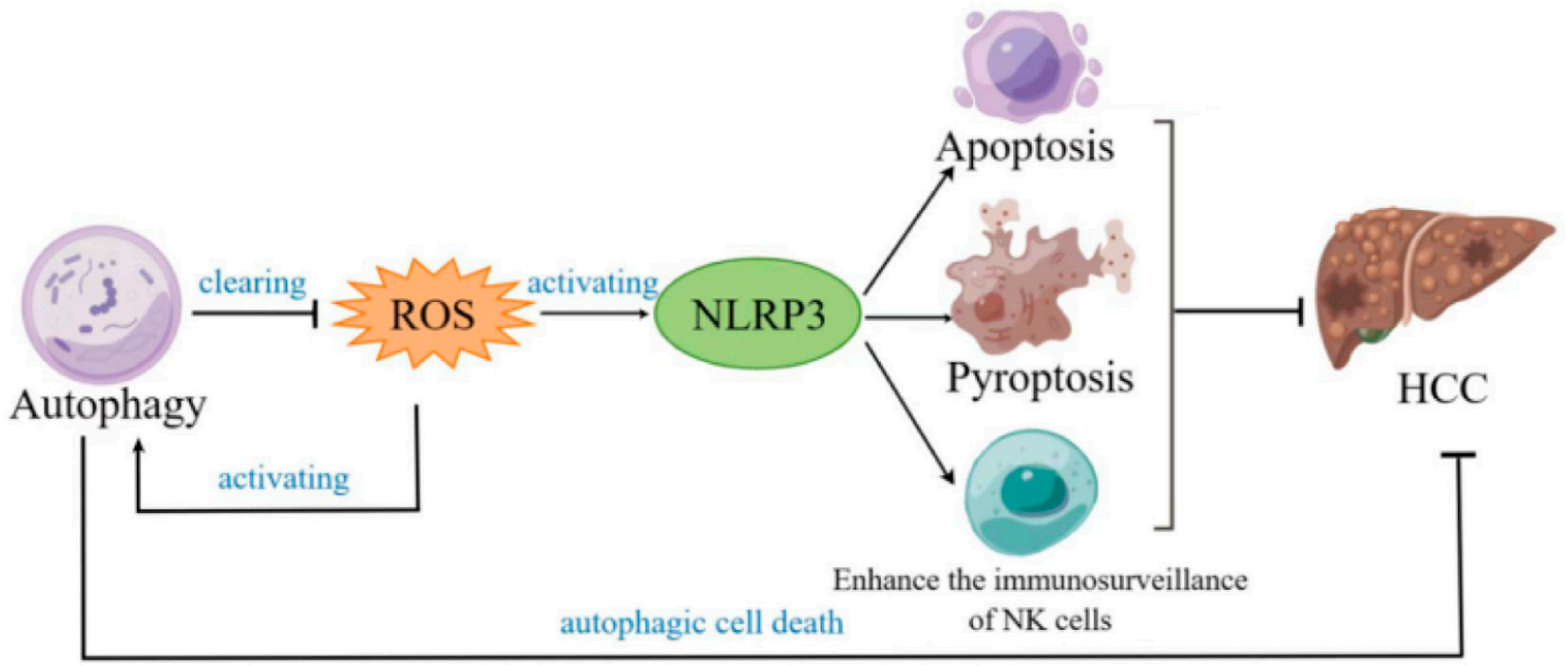
Diagram of the role of autophagy, NLRP3 and reactive oxygen species (ROS) in hepatocellular carcinoma.

The role of NLRP3 inflammasome in HCC remains controversial. It has been reported that NLRP3 inflammasome plays a dual role in cancer (Moossavi et al., 2018; Hamarsheh and Zeiser, 2020; Sharma and Kanneganti, 2021). In the literature cited in this review, sometimes NLRP3 inflammasome plays a role in inhibiting HCC, and sometimes it plays the opposite role. The reason may be related to the different HCC cell line and the different course of HCC. As the core part of inflammation, the activation of NLRP3 inflammasome promotes the release of IL-1β and IL-18, thereby accelerating the growth and metastasis of tumors. On the contrary, when the tissue is damaged to a certain extent, the activation of inflammasome inhibits tumor cells by promoting pyroptosis and apoptosis. The conditions under which NLRP3 inflammasomes are beneficial to HCC and inhibit HCC need to be clarified by future researches. In addition, it can be seen from this review that NLRP3 inflammasome inhibits HCC through inducing pyroptosis, apoptosis, cell cycle arrest and the enhancement of the immunosurveillance of NK cells. Moreover, autophagy and ROS play a role in HCC by associating with NLRP3 inflammasome (Figure 2). However, the mechanism of autophagy and ROS remains unclear and needs further study. At present, there are few studies on the role of NLRP3 inflammasome in cancer by regulating NK. Therefore, how NLRP3 inflammasome regulates NK cells in HCC is a topic worthy of study.

It is believed that with the deepening of research, ROS/NLRP3 inflammasome will provide an important target for the treatment of HCC.
